# Supplementary material for: Results of a large scale study of the binding of 50 type II inhibitors to 348 kinases: The role of protein reorganization
Source: bioRxiv. 2026 Feb 8:2026.02.05.704068. Preprint. [Version 1] doi: 10.64898/2026.02.05.704068 (PMC12889613; doi:10.64898/2026.02.05.704068)
Supplement: Supplement 1 [file media-1.pdf]

Supplementary Material

Performance of the Boltz-2 structure-based Machine Learning model

Protein-ligand interaction prediction models can be broadly categorized into two classes: sequence- based models and structure-based models [1]. In section 8, we introduced the sequence-based framework DeepDTA-Gen [2] and evaluated its predictive power on the Davis training dataset and the Schrödinger unseen dataset. In the present section, we shift focus to structure-based approaches and examine Boltz-2 [3], a co-folding model designed to predict three-dimensional molecular structures and binding affinities of protein-ligand complexes, as well as complexes involving DNA and RNA.

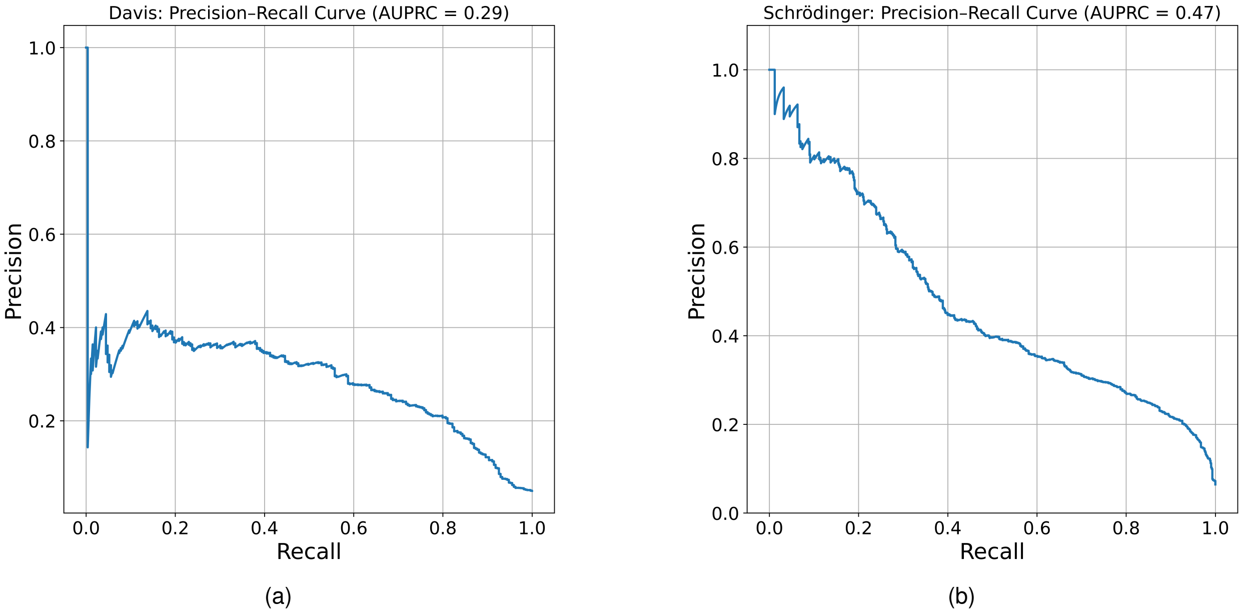

Figure 1: Precision-Recall curve evaluation for binary binding prediction $K_d < 100$ nM on a) the 16 type II inhibitors from Davis dataset and b) 34 type II inhibitors from the Schrödinger dataset.

The performance of Boltz-2 was evaluated using 16 type II inhibitors from the Davis dataset Fig. 1 (a) and 34 Type II inhibitors from the Schrödinger dataset Fig. 1(b), following the same evaluation protocol used for DeepDTAGen. Boltz-2 achieved an AUPRC of 0.28 on the Davis dataset, compared to 0.46 on the Schrödinger dataset. These results indicate poor overall predictive performance, with limited discrimination beyond baseline expectations across both datasets.

Inhibitors

Table S1: List of 50 type II kinase inhibitors. The table provides the inhibitor index, name, Number of hits (across 348-kinase panel), year of publication, and the corresponding reference. Imatinib, the first developed inhibitor in this list (highlighted in the 38th row), is included as a reference point.

#	Inhibitor	Number of hits	Year	Citation
1	Olverembatinib	229	2013	[4]
2	Ponatinib	224	2010	[5, 6]
3	AST-487	206	2007	[7]

Continued on next page

Table S1

#	Inhibitor	Number of hits	Year	Citation
4	EXEL-2880|GSK-1363089	188	2005	[8]
5	Rebastinib	181	2011	[9]
6	NG25	155	2014	[10]
7	RIPK1-IN-4	145	2013	[11]
8	Golvatinib	133	2009	[12]
9	LY3009120	123	2015	[13]
10	MAPK13-IN-1	111	2012	[14]
11	Tovorafenib	109	2017	[15]
12	Merestinib	101	2013	[16]
13	Sorafenib	100	2001	[17]
14	Linifanib (ABT-869)	88	2007	[18]
15	PF-6683324	77	2018	[19]
16	PLX-4720	74	2008	[20]
17	Bafetinib	71	2007	[21]
18	BIRB-796	71	2002	[22]
19	Pexidartinib	68	2004	[23]
20	DDR1-IN-1	66	2013	[24]
21	NVP-BHG712	65	2016	[25]
22	CHIR-265|RAF-265	65	2006	[26, 27]
23	TAK-632	65	2013	[28]
24	AZD-1152HQPA	62	2007	[29, 30]
25	Ki-20227	62	2006	[31]
26	DDR Inhibitor	60	2015	[32]
27	Nilotinib	52	2007	[33]
28	GNE-9815	51	2021	[34]
29	Belvarafenib	48	2021	[35]
30	p38-a MAPK-IN-1	45	2009	[36]
31	AMG-706	42	2007	[37]
32	AWL-II-38.3	40	2009	[38]
33	AAL993	39	2015	[39]
34	ALW-II-49-7	39	2009	[38]
35	MLN-518	37	2002	[40]
36	AC220	36	2009	[41]
37	RAF709	32	2017	[42]

Continued on next page

Table S1

#	Inhibitor	Number of hits	Year	Citation
38	NVP-BHG712 isomer	32	2016	[25]
39	Imatinib	29	1996	[43, 44]
40	PDGFRa kinase inhibitor 1	28	2017	[45]
41	GSK2334470	23	2011	[46]
42	AB-1010	23	2008	[47]
43	Exarafenib	21	2024	[48]
44	B-Raf IN 1	21	2009	[49]
45	Naporafenib	21	2019	[50]
46	SR-318	14	2019	[51]
47	DDR1-IN-4	9	2018	[52]
48	GW-2580	4	2006	-
49	MP7	3	2011	[53]
50	TH470	3	2022	[54]

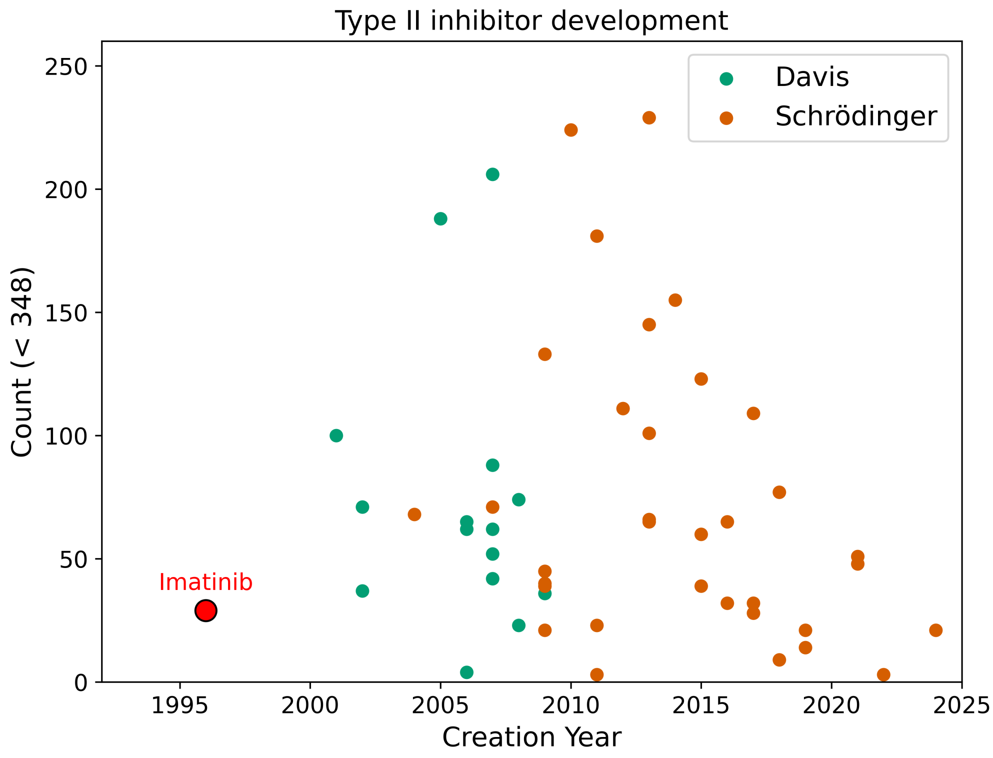

Figure S1: Selectivity of type II kinase inhibitors over time. The x-axis indicates the year of development, and the y-axis shows inhibitor selectivity (number of kinases bound out of a 348-kinase panel). Imatinib, the first type II inhibitor developed, is shown explicitly. Data points are color-coded: green for inhibitors reported in Davis et al. and brown for inhibitors reported in Schrödinger.

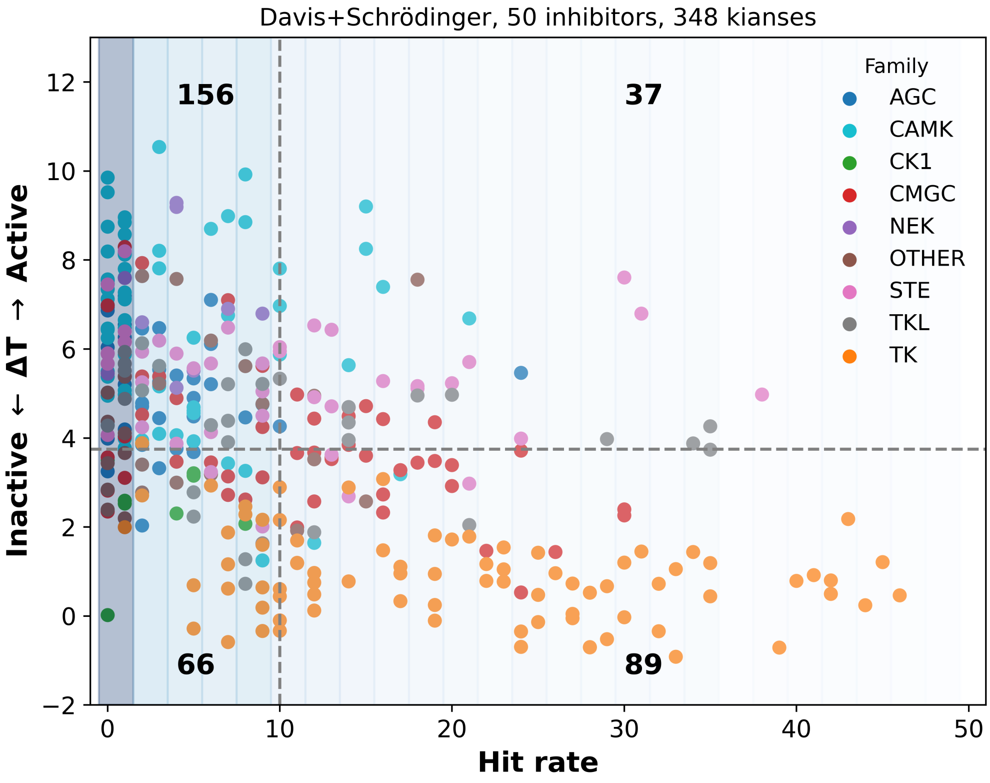

Figure S2: Selectivity landscape constructed using the new contact difference map (CDM) for three kinase families: CMGC, STE and CAMK. The vertical dashed line indicates the selectivity barrier, separating selective kinases from promiscuous ones. The horizontal dashed line represents the optimal threading-score threshold determined from the ROC analysis (Fig. 3), distinguishing kinases predicted to be promiscuous from those classified as selective. Numbers within each square denote the count of kinases falling into that region. Vertical color bars indicate the density of kinases per bin, with darker shading corresponding to bins containing more kinases.

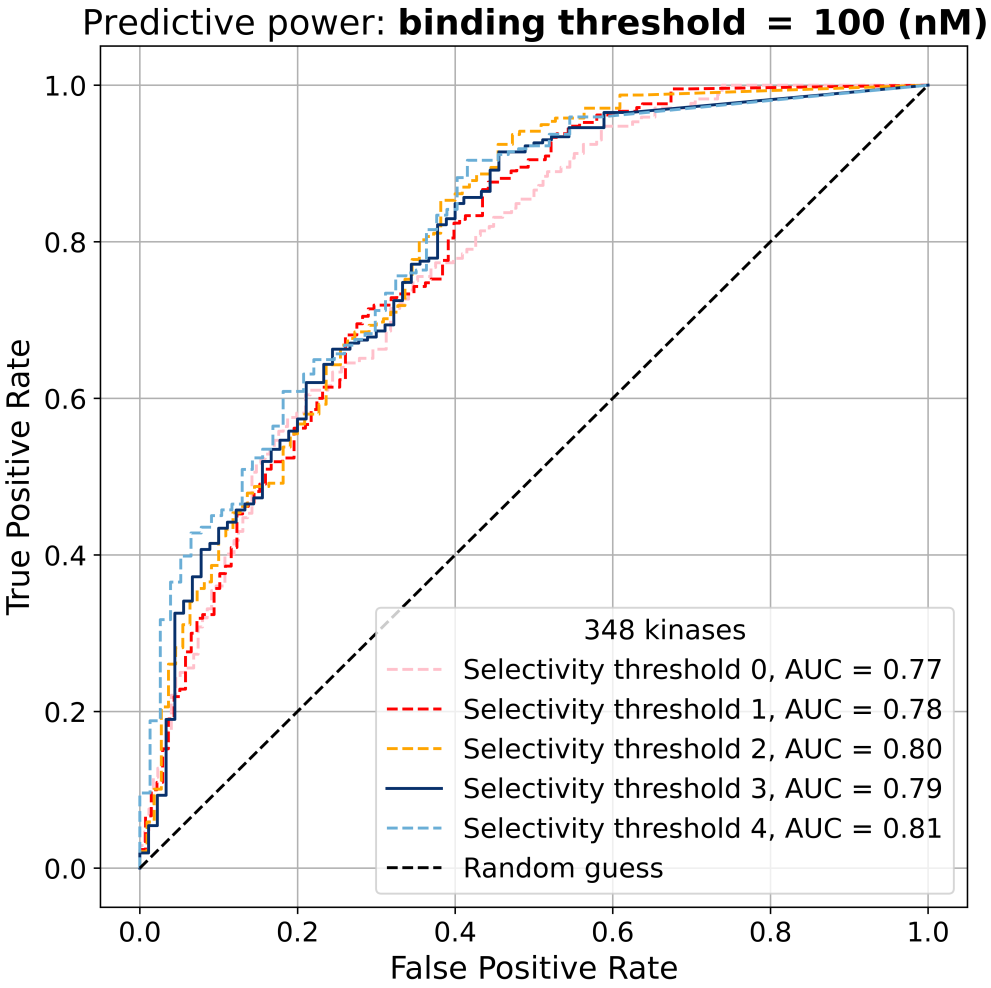

Figure S3: ROC curves assessing the ability of the threading score to predict kinase selectivity at selectivity thresholds of 0,1,2,3 and 4. Complete set of 348 kinases evaluated using the $K_d < 100$ nM hit definition.

References

[1] Yunjiang Zhang, Shuyuan Li, Kong Meng, and Shaorui Sun. Machine learning for sequence and structure-based protein–ligand interaction prediction. *Journal of chemical information and modeling*, 64(5):1456–1472, 2024.

[2] Pir Masoom Shah, Huimin Zhu, Zhangli Lu, Kaili Wang, Jing Tang, and Min Li. Deepdtagen: a multitask deep learning framework for drug-target affinity prediction and target-aware drugs generation. *Nature Communications*, 16(1):5021, 2025.

[3] Saro Passaro, Gabriele Corso, Jeremy Wohlwend, Mateo Reveiz, Stephan Thaler, Vignesh Ram Somnath, Noah Getz, Tally Portnoi, Julien Roy, Hannes Stark, et al. Boltz-2: Towards accurate and efficient binding affinity prediction. *BioRxiv*, 2025.

[4] Xiaomei Ren, Xiaofen Pan, Zhang Zhang, Deping Wang, Xiaoyun Lu, Yupeng Li, Donghai Wen, Huoyou Long, Jinfeng Luo, Yubing Feng, Xiaoxi Zhuang, Fengxiang Zhang, Jianqi Liu, Fang Leng, Xingfen Lang, Yang Bai, Miaoqin She, Zhengchao Tu, Jingxuan Pan, and Ke Ding. Identification of gzd824 as an orally bioavailable inhibitor that targets phosphorylated and nonphosphorylated breakpoint cluster region-abelson (bcr-abl) kinase and overcomes clinically acquired mutation-induced resistance against imatinib. *Journal of Medicinal Chemistry*, 56:879–894, 2 2013.

[5] Tianjun Zhou, Lois Commodore, Wei Sheng Huang, Yihan Wang, Mathew Thomas, Jeff Keats, Qihong Xu, Victor M. Rivera, William C. Shakespeare, Tim Clackson, David C. Dalgarno, and Xiaotian Zhu. Structural mechanism of the pan-bcr-abl inhibitor ponatinib (ap24534): Lessons for overcoming kinase inhibitor resistance. *Chemical Biology and Drug Design*, 77:1–11, 1 2011.

[6] Wei Sheng Huang, Chester A. Metcalf, Raji Sundaramoorthi, Yihan Wang, Dong Zou, R. Mathew Thomas, Xiaotian Zhu, Lisi Cai, David Wen, Shuangying Liu, Jan Romero, Jiwei Qi, Ingrid Chen, Geetha Banda, Scott P. Lentini, Sasmita Das, Qihong Xu, Jeff Keats, Frank Wang, Scott Wardwell, Yaoyu Ning, Joseph T. Snodgrass, Marc I. Broudy, Karin Russian, Tianjun Zhou, Lois Commodore, Narayana I. Narasimhan, Qurish K. Mohemmad, John Iuliucci, Victor M. Rivera, David C. Dalgarno, Tomi K. Sawyer, Tim Clackson, and William C. Shakespeare. Discovery of 3-[2-(imidazo[1,2- b]pyridazin-3-yl)ethynyl]-4-methyl- n -{4-[(4-methylpiperazin-1-yl)methyl]-3-(trifluoromethyl)phenyl}benzamide (ap24534), a potent, orally active pan-inhibitor of breakpoint cluster region-abelson (bcr-abl) kinase including the t315i gatekeeper mutant. *Journal of Medicinal Chemistry*, 53:4701–4719, 6 2010.

[7] Nagako Akeno-Stuart, Michelle Croyle, Jeffrey A. Knauf, Roberta Malaguarnera, Donata Vitagliano, Massimo Santoro, Christine Stephan, Konstantina Grosios, Markus Wartmann, Robert Cozens, Giorgio Caravatti, Doriano Fabbro, Heidi A. Lane, and James A. Fagin. The ret kinase inhibitor nvp-ast487 blocks growth and calcitonin gene expression through distinct mechanisms in medullary thyroid cancer cells. *Cancer Research*, 67:6956–6964, 7 2007.

[8] Fawn Qian, Stefan Engst, Kyoko Yamaguchi, Yu Peiwen, Kwang Ai Won, Lillian Mock, Tracy Lou, Jenny Tan, Connie Li, Danny Tam, Julie Lougheed, F. Michael Yakes, Frauke Bentzien, Xu Wei, Tal Zaks, Richard Wooster, Joel Greshock, and Alison H. Joly. Inhibition of tumor cell growth, invasion, and metastasis by exel-2880 (xl880, gsk1363089), a novel inhibitor of hgf and vegf receptor tyrosine kinases. *Cancer Research*, 69:8009–8016, 10 2009.

[9] Wayne W. Chan, Scott C. Wise, Michael D. Kaufman, Yu Mi Ahn, Carol L. Ensinger, Torsten Haack, Molly M. Hood, Jennifer Jones, John W. Lord, Wei Ping Lu, David Miller, William C. Patt, Bryan D. Smith, Peter A. Petillo, Thomas J. Rutkoski, Hanumaiah Telikepalli, Lakshminarayana Vogeti, Tony Yao, Lawrence Chun, Robin Clark, Peter Evangelista, L. Cristina Gavrilescu, Katherine Lazarides, Virginia M. Zaleskas, Lance J. Stewart, Richard A. Van Etten, and Daniel L. Flynn. Conformational control inhibition of the bcr-abl1 tyrosine kinase, including the gatekeeper t315i mutant, by the switch-control inhibitor dcc-2036. *Cancer Cell*, 19:556–568, 2011.

[10] Li Tan, Tyzoon Nomanbhoy, Deepak Gurbani, Matthew Patricelli, John Hunter, Jiefei Geng, Lina Herhaus, Jianming Zhang, Eduardo Pauls, Youngjin Ham, et al. Discovery of type ii inhibitors of tgfβ-activated kinase 1 (tak1) and mitogen-activated protein kinase kinase kinase kinase 2 (map4k2). *Journal of medicinal chemistry*, 58(1):183–196, 2015.

[11] Philip A. Harris, Deepak Bandyopadhyay, Scott B. Berger, Nino Campobasso, Carol A. Capriotti, Julie A. Cox, Lauren Dare, Joshua N. Finger, Sandra J. Hoffman, Kirsten M. Kahler, Ruth Lehr, John D. Lich, Rakesh Nagilla, Robert T. Nolte, Michael T. Ouellette, Christina S. Pao, Michelle C. Schaeffer, Angela Smallwood, Helen H. Sun, Barbara A. Swift, Rachel D. Totoritis, Paris Ward, Robert W. Marquis, John Bertin, and Peter J. Gough. Discovery of small molecule rip1 kinase inhibitors for the treatment of pathologies associated with necroptosis. *ACS Medicinal Chemistry Letters*, 4:1238–1243, 12 2013.

[12] Takayuki Nakagawa, Osamu Tohyama, Atsumi Yamaguchi, Tomohiro Matsushima, Keiko Takahashi, Setsuo Funasaka, Shuji Shirotori, Makoto Asada, and Hiroshi Obaishi. E7050: A dual c-met and vegfr-2 tyrosine kinase inhibitor promotes tumor regression and prolongs survival in mouse xenograft models. *Cancer Science*, 101:210–215, 1 2010.

[13] James R. Henry, Michael D. Kaufman, Sheng-Bin Peng, Yu Mi Ahn, Timothy M. Caldwell, Lakshminarayana Vogeti, Hanumaiah Telikepalli, Wei-Ping Lu, Molly M. Hood, Thomas J. Rutkoski, Bryan D. Smith, Subha Vogeti, David Miller, Scott C. Wise, Lawrence Chun, Xiaoyi Zhang, Youyan Zhang, Lisa Kays, Philip A. Hipskind, Aaron D. Wrobleski, Karen L. Lobb, Julia M. Clay, Jeffrey D. Cohen, Jennie L. Walgren, Denis McCann, Phenil Patel, David K. Clawson, Sherry Guo, Danalyn Manglicmot, Chris Groshong, Cheyenne Logan, James J. Starling, and Daniel L. Flynn. Discovery of 1-(3,3-dimethylbutyl)-3-(2-fluoro-4-methyl-5-(7-methyl-2-(methylamino)pyrido[2,3-d]pyrimidin-6-yl)phenyl)urea (ly3009120) as a pan-raf inhibitor with minimal paradoxical activation and activity against braf or ras mutant tumor cells. *Journal of Medicinal Chemistry*, 58(10):4165–4179, 2015.

[14] Yael G. Alevy, Anand C. Patel, Arthur G. Romero, Dhara A. Patel, Jennifer Tucker, William T. Roswit, Chantel A. Miller, Richard F. Heier, Derek E. Byers, Tom J. Brett, and Michael J. Holtzman. Il-13-induced airway mucus production is attenuated by mapk13 inhibition. *Journal of Clinical Investigation*, 122:4555–4568, 12 2012.

[15] Yu Sun, John A. Alberta, Catherine Pilarz, David Calligaris, Emily J. Chadwick, Shakti H. Ramkissoon, Lori A. Ramkissoon, Veronica Matia Garcia, Emanuele Mazzola, Liliana Goumnerova, Michael Kane, Zhan Yao, Mark W. Kieran, Keith L. Ligon, William C. Hahn, Levi A. Garraway, Neal Rosen, Nathanael S. Gray, Nathalie Y. Agar, Sara J. Buhrlage, Rosalind A. Segal, and Charles D. Stiles. A brain-penetrant raf dimer antagonist for the noncanonical braf oncoprotein of pediatric low-grade astrocytomas. *Neuro-Oncology*, 19:774–785, 2017.

[16] S. Betty Yan, Victoria L. Peek, Rose Ajamie, Sean G. Buchanan, Jeremy R. Graff, Steven A. Heidler, Yu Hua Hui, Karen L. Huss, Bruce W. Konicek, Jason R. Manro, Chuan Shih, Julie A. Stewart, Trent R. Stewart, Stephanie L. Stout, Mark T. Uhlik, Suzane L. Um, Yong Wang, Wenjuan Wu, Lei Yan, Wei J. Yang, Boyu Zhong, and Richard A. Walgren. Ly2801653 is an orally bioavailable multi-kinase inhibitor with potent activity against met, mst1r, and other oncoproteins, and displays anti-tumor activities in mouse xenograft models. *Investigational New Drugs*, 31:833–844, 8 2013.

[17] J F Lyons, S Wilhelm, B Hibner, and G Bollag. Discovery of a novel raf kinase inhibitor ras epidemiology. Technical report, 2001.

[18] Yujia Dai, Kresna Hartandi, Zhiqin Ji, Asma A Ahmed, Daniel H Albert, Joy L Bauch, Jennifer J Bouska, Peter F Bousquet, George A Cunha, Keith B Glaser, et al. Discovery of n-(4-(3-amino-1 h-indazol-4-yl) phenyl)-n ‘-(2-fluoro-5-methylphenyl) urea (abt-869), a 3-aminoindazole-based orally active multitargeted receptor tyrosine kinase inhibitor. *Journal of medicinal chemistry*, 50(7):1584–1597, 2007.

[19] Luping Qiu, Kymberly Levine, Ketan S. Gajiwala, Ciarán N. Cronin, Asako Nagata, Eric Johnson, Michelle Kraus, John Tatlock, Robert Kania, Timothy Foley, and Shaoxian Sun. Small molecule inhibitors reveal ptk6 kinase is not an oncogenic driver in breast cancers. *PLoS ONE*, 13, 6 2018.

[20] James Tsai, John T Lee, Weiru Wang, Jiazhong Zhang, Hanna Cho, Shumeye Mamo, Ryan Bremer, Sam Gillette, Jun Kong, Nikolas K Haass, et al. Discovery of a selective inhibitor of oncogenic b-raf kinase with potent antimelanoma activity. *Proceedings of the National Academy of Sciences*, 105(8):3041–3046, 2008.

[21] Tatsuya Horio, Tomohiro Hamasaki, Toshihiko Inoue, Tatsushi Wakayama, Shinsaku Itou, Haruna Naito, Tetsuo Asaki, Hiroki Hayase, and Tomoko Niwa. Structural factors contributing to the abl/lyn dual inhibitory activity of 3-substituted benzamide derivatives. *Bioorganic and Medicinal Chemistry Letters*, 17:2712–2717, 5 2007.

[22] Christopher Pargellis, Liang Tong, Laurie Churchill, Pier F. Cirillo, Thomas Gilmore, Anne G. Graham, Peter M. Grob, Eugene R. Hickey, Neil Moss, Susan Pav, and John Regan. Inhibition of p38 map kinase by utilizing a novel allosteric binding site. *Nature Structural Biology*, 9:268–272, 2002.

[23] Teresa S. Kim, Michael J. Cavnar, Noah A. Cohen, Eric C. Sorenson, Jonathan B. Greer, Adrian M. Seifert, Megan H. Crawley, Benjamin L. Green, Rachel Popow, Nagavarakishore Pillarsetty, Darren R. Veach, Anson T. Ku, Ferdinand Rossi, Peter Besmer, Cristina R. Antonescu, Shan Zeng, and Ronald P. De Matteo. Increased kit inhibition enhances therapeutic efficacy in gastrointestinal stromal tumor. *Clinical Cancer Research*, 20:2350–2362, 5 2014.

[24] Hyung Gu Kim, Li Tan, Ellen L. Weisberg, Feiyang Liu, Peter Canning, Hwan Geun Choi, Scott A. Ezell, Hong Wu, Zheng Zhao, Jinhua Wang, Anna Mandinova, James D. Griffin, Alex N. Bullock, Qingsong Liu, Sam W. Lee, and Nathanael S. Gray. Discovery of a potent and selective ddr1 receptor tyrosine kinase inhibitor. *ACS Chemical Biology*, 8:2145–2150, 10 2013.

[25] Alix Tröster, Stephanie Heinzlmeir, Benedict Tilman Berger, Santosh L. Gande, Krishna Saxena, Sridhar Sreeramulu, Verena Linhard, Amir H. Nasiri, Michael Bolte, Susanne Müller, Bernhard Kuster, Guillaume Médard, Denis Kudlinzki, and Harald Schwalbe. Nvp-bhg712: Effects of regioisomers on the affinity and selectivity toward the ephrin family. *ChemMedChem*, 13:1629–1633, 8 2018.

[26] Clara Montagut and Jeff Settleman. Targeting the raf-mek-erk pathway in cancer therapy, 10 2009.

[27] Teresa E. Williams, Sharadha Subramanian, Joelle Verhagen, Christopher M. McBride, Abran Costales, Leonard Sung, William Antonios-Mccrea, Maureen McKenna, Alicia K. Louie, Savithri Ramurthy, Barry Levine, Cynthia M. Shafer, Timothy MacHajewski, Paul A. Renhowe, Brent A. Appleton, Payman Amiri, James Chou, Darrin Stuart, Kimberly Aardalen, and Daniel Poon. Discovery of raf265: A potent mut-b-raf inhibitor for the treatment of metastatic melanoma. *ACS Medicinal Chemistry Letters*, 6:961–965, 9 2015.

[28] Masanori Okaniwa, Masaaki Hirose, Takeo Arita, Masato Yabuki, Akito Nakamura, Terufumi Takagi, Tomohiro Kawamoto, Noriko Uchiyama, Akihiko Sumita, Shunichirou Tsutsumi, Tsuneaki Tottori, Yoshitaka Inui, Bi Ching Sang, Jason Yano, Kathleen Aertgeerts, Sei Yoshida, and Tomoyasu Ishikawa. Discovery of a selective kinase inhibitor (tak-632) targeting pan-raf inhibition: Design, synthesis, and biological evaluation of c -7-substituted 1,3-benzothiazole derivatives. *Journal of Medicinal Chemistry*, 56:6478–6494, 8 2013.

[29] Robert W. Wilkinson, Rajesh Odedra, Simon P. Heaton, Stephen R. Wedge, Nicholas J. Keen, Claire Crafter, John R. Foster, Madeleine C. Brady, Alison Bigley, Elaine Brown, Kate F. Byth, Nigel C. Barrass, Kirsten E. Mundt, Kevin M. Foote, Nicola M. Heron, Frederic H. Jung, Andrew A. Mortlock, F. Thomas Boyle, and Stephen Green. Azd1152, a selective inhibitor of aurora b kinase, inhibits human tumor xenograft growth by inducing apoptosis. *Clinical Cancer Research*, 13:3682–3688, 6 2007.

[30] Andrew A. Mortlock, Kevin M. Foote, Nicola M. Heron, Frédéric H. Jung, Georges Pasquet, Jean Jacques M. Lohmann, Nicolas Warin, Fabrice Renaud, Chris De Savi, Nicola J. Roberts, Trevor Johnson, Cyril B. Dousson, George B. Hill, David Perkins, Glenn Hatter, Robert W. Wilkinson, Stephen R. Wedge, Simon P. Heaton, Rajesh Odedra, Nicholas J. Keen, Claire Crafter, Elaine Brown, Katherine Thompson, Stephen Brightwell, Liz Khatri, Madeleine C. Brady, Sarah Kearney, David McKillop, Steve Rhead, Tony Parry, and Stephen Green. Discovery, synthesis, and in vivo activity of a new class of pyrazoloquinazolines as selective inhibitors of aurora b kinase. *Journal of Medicinal Chemistry*, 50:2213–2224, 5 2007.

[31] Hiroaki Ohno, Kazuo Kubo, Hideko Murooka, Yoshiko Kobayashi, Tsuyoshi Nishitoba, Masabumi Shibuya, Toshiyuki Yoneda, and Toshiyuki Isoe. A c-fms tyrosine kinase inhibitor, ki20227,suppresses osteoclast differentiation and osteolytic bone destruction in a bone metastasis model. *Molecular Cancer Therapeutics*, 5:2634–2643, 11 2006.

[32] Christopher W. Murray, Valerio Berdini, Ildiko M. Buck, Maria E. Carr, Anne Cleasby, Joseph E. Coyle, Jayne E. Curry, James E.H. Day, Phillip J. Day, Keisha Hearn, Aman Iqbal, Lydia Y.W. Lee, Vanessa Martins, Paul N. Mortenson, Joanne M. Munck, Lee W. Page, Sahil Patel, Susan Roomans, Kirsten Smith, Emiliano Tamanini, and Gordon Saxty. Fragment-based discovery of potent and selective ddr1/2 inhibitors. *ACS Medicinal Chemistry Letters*, 6:798–803, 7 2015.

[33] Ellen Weisberg, Paul W. Manley, Werner Breitenstein, Josef Brüggen, Sandra W. Cowan-Jacob, Arghya Ray, Brian Huntly, Doriano Fabbro, Gabriele Fendrich, Elizabeth Hall-Meyers, Andrew L. Kung, Jürgen

Mestan, George Q. Daley, Linda Callahan, Laurie Catley, Cara Cavazza, Azam Mohammed, Donna Neuberg, Renee D. Wright, D. Gary Gilliland, and James D. Griffin. Characterization of amn107, a selective inhibitor of native and mutant bcr-abl. *Cancer Cell*, 7:129–141, 2005.

[34] Malcolm P. Huestis, Matthew R. Durk, Charles Eigenbrot, Paul Gibbons, Thomas L. Hunsaker, Hank La, Dennis H. Leung, Wendy Liu, Shiva Malek, Mark Merchant, John G. Moffat, Christine S. Muli, Christine J. Orr, Brendan T. Parr, Frances Shanahan, Christopher J. Sneeringer, Weiru Wang, Ivana Yen, Jianping Yin, Joachim Rudolph, and Michael Siu. Targeting kras mutant cancers via combination treatment: Discovery of a pyridopyridazinone pan-raf kinase inhibitor. *ACS Medicinal Chemistry Letters*, 12:791–797, 5 2021.

[35] Ivana Yen, Frances Shanahan, Jeeyun Lee, Yong Sang Hong, Sang Joon Shin, Amanda R. Moore, Jawahar Sudhamsu, Matthew T. Chang, Inhwan Bae, Darlene Dela Cruz, Thomas Hunsaker, Christiaan Klijn, Nicholas P.D. Liau, Eva Lin, Scott E. Martin, Zora Modrusan, Robert Piskol, Ehud Segal, Avinashnarayan Venkatanarayan, Xin Ye, Jianping Yin, Liangxuan Zhang, Jin Soo Kim, Hyeong Seok Lim, Kyu Pyo Kim, Yu Jung Kim, Hye Sook Han, Soo Jung Lee, Seung Tae Kim, Minkyu Jung, Yoon hee Hong, Young Su Noh, Munjeong Choi, Oakpil Han, Malgorzata Nowicka, Shrividhya Srinivasan, Yibing Yan, Tae Won Kim, and Shiva Malek. Araf mutations confer resistance to the raf inhibitor belvarafenib in melanoma. *Nature*, 594:418–423, 6 2021.

[36] Sabine Klüter, Christian Grütter, Tabassum Naqvi, Matthias Rabiller, Jeffrey R. Simard, Vijaykumar Pawar, Matthäus Getlik, and Daniel Rauh. Displacement assay for the detection of stabilizers of inactive kinase conformations. *Journal of Medicinal Chemistry*, 53:357–367, 2010.

[37] Angela Coxon, James Bready, Paul Hughes, Juan Estrada, Ling Wang, Thomas DeMelfi, Nicholas Doerr, Stephen Kaufman, Robert Radinsky, Richard Kendall, et al. Motesanib diphosphate (amg 706) inhibits the growth of medullary thyroid carcinoma in a nude mouse model. *Cancer Research*, 67(9_Supplement):LB–283, 2007.

[38] Yongmun Choi, Farisa Syeda, John R. Walker, Patrick J. Finerty, Dominic Cuerrier, Amy Wojciechowski, Qingsong Liu, Sirano Dhe-Paganon, and Nathanael S. Gray. Discovery and structural analysis of eph receptor tyrosine kinase inhibitors. *Bioorganic and Medicinal Chemistry Letters*, 19:4467–4470, 8 2009.

[39] Guido Bold, Christian Schnell, Pascal Furet, Paul McSheehy, Josef Brüggen, Jürgen Mestan, Paul W. Manley, Peter Drückes, Marion Burglin, Ursula Dürler, Jacqueline Loretan, Robert Reuter, Markus Wartmann, Andreas Theuer, Beatrice Bauer-Probst, Georg Martiny-Baron, Peter Allegrini, Arnaud Goepfert, Jeanette Wood, and Amanda Littlewood-Evans. A novel potent oral series of vegfr2 inhibitors abrogate tumor growth by inhibiting angiogenesis. *Journal of Medicinal Chemistry*, 59:132–146, 1 2016.

[40] Louise M Kelly, Jin-Chen Yu, Christina L Boulton, Mutiah Apatira, Jason Li, Carol M Sullivan, Ifor Williams, Sonia M Amaral, David P Curley, Nicole Duclos, et al. Ct53518, a novel selective flt3 antagonist for the treatment of acute myelogenous leukemia (aml). *Cancer cell*, 1(5):421–432, 2002.

[41] Qi Chao, Kelly G Sprankle, Robert M Grotzfeld, Andiliy G Lai, Todd A Carter, Anne Marie Velasco, Ruwanthi N Gunawardane, Merryl D Cramer, Michael F Gardner, Joyce James, et al. Identification of n-(5-tert-butyl-isoxazol-3-yl)-n-4-[7-(2-morpholin-4-yl-ethoxy) imidazo [2, 1-b][1, 3] benzothiazol-2-yl] phenyl urea dihydrochloride (ac220), a uniquely potent, selective, and efficacious fms-like tyrosine kinase-3 (flt3) inhibitor. *Journal of medicinal chemistry*, 52(23):7808–7816, 2009.

[42] Gisele A Nishiguchi, Alice Rico, Huw Tanner, Robert J Aversa, Benjamin R Taft, Sharadha Subramanian, Lina Setti, Matthew T Burger, Lifeng Wan, Victoriano Tamez, et al. Design and discovery of n-(2-methyl-5-morpholino-6-((tetrahydro-2 h-pyran-4-yl) oxy)-[3, 3-bipyridin]-5-yl)-3-(trifluoromethyl) benzamide (raf709): A potent, selective, and efficacious raf inhibitor targeting ras mutant cancers. *Journal of Medicinal Chemistry*, 60(12):4869–4881, 2017.

[43] J rg Zimmermann, Elisabeth Buchdunger, Helmut Mett, Thomas Meyer, and Nicholas B Lydon. Potent and selective inhibitors of the abl-kinase: Phenylamino-pyrimidine (pap) derivatives. Technical report, 1997.

[44] Thomas Schindler, William Bornmann, Patricia Pellicena, W Todd Miller, Bayard Clarkson, and John Kuriyan. Structural mechanism for sti-571 inhibition of abelson tyrosine kinase. *Science*, 289(5486):1938–1942, 2000.

[45] Qiang Wang, Feiyang Liu, Shuang Qi, Ziping Qi, Xiao E. Yan, Beilei Wang, Aoli Wang, Wei Wang, Cheng Chen, Xiaochuan Liu, Zongru Jiang, Zhenquan Hu, Li Wang, Wenchao Wang, Tao Ren, Shanchun Zhang, Cai Hong Yun, Qingsong Liu, and Jing Liu. Discovery of 4-((n-(2-(dimethylamino)ethyl)acrylamido)methyl)-n-(4-methyl-3-((4-(pyridin-3-yl)pyrimidin-2-yl)amino)phenyl)benzamide (chmfl-pdgfr-159) as a highly selective type ii pdgfrα kinase inhibitor for pdgfrα driving chronic eosinophilic leukemia. *European Journal of Medicinal Chemistry*, 150:366–384, 4 2018.

[46] Ayaz Najafov, Eeva M. Sommer, Jeffrey M. Axten, M. Phillip DeYoung, and Dario R. Alessi. Characterization of gsk2334470, a novel and highly specific inhibitor of pdk1. *Biochemical Journal*, 433:357–369, 1 2011.

[47] Kevin A Hahn, Greg Oglivie, Tony Rusk, Patrice Devauchelle, Amy Leblanc, Alfred Legendre, Barbara Powers, Phillip S Leventhal, J-P Kinet, Fabienne Palmerini, et al. Masitinib is safe and effective for the treatment of canine mast cell tumors. *Journal of Veterinary Internal Medicine*, 22(6):1301–1309, 2008.

[48] Young K. Chen, Toufike Kanouni, Lee D. Arnold, Jason M. Cox, Elisabeth Gardiner, Kathryn Grandinetti, Ping Jiang, Stephen W. Kaldor, Catherine Lee, Chun Li, Eric S. Martin, Nichol Miller, Eric A. Murphy, Noel Timple, John S. Tyhonas, Angie Vassar, Tim S. Wang, Richard Williams, Ding Yuan, and Robert S. Kania. The discovery of exarafenib (kin-2787): Overcoming the challenges of pan-raf kinase inhibition. *Journal of Medicinal Chemistry*, 67:1747–1757, 2 2024.

[49] Adrian L. Smith, Frenel F. DeMorin, Nick A. Paras, Qi Huang, Jeffrey K. Petkus, Elizabeth M. Doherty, Thomas Nixey, Joseph L. Kim, Douglas A. Whittington, Linda F. Epstein, Matthew R. Lee, Mark J. Rose, Carol Babij, Manory Fernando, Kristen Hess, Quynh Le, Pedro Beltran, and Josette Carnahan. Selective inhibitors of the mutant b-raf pathway: Discovery of a potent and orally bioavailable aminoisoquinoline. *Journal of Medicinal Chemistry*, 52:6189–6192, 10 2009.

[50] Savithri Ramurthy, Benjamin R. Taft, Robert J. Aversa, Paul A. Barsanti, Matthew T. Burger, Yan Lou, Gisele A. Nishiguchi, Alice Rico, Lina Setti, Aaron Smith, Sharadha Subramanian, Victoriano Tamez, Huw Tanner, Lifeng Wan, Cheng Hu, Brent A. Appleton, Mulugeta Mamo, Laura Tandeske, John E. Tellew, Shenlin Huang, Qin Yue, Apurva Chaudhary, Hung Tian, Raman Iyer, A. Quamrul Hassan, Lesley A. Mathews Griner, Laura R. La Bonte, Vesselina G. Cooke, Anne Van Abbema, Hanne Merritt, Kalyani Gampa, Fei Feng, Jing Yuan, Yuji Mishina, Yingyun Wang, Jacob R. Haling, Sepideh Vaziri, Mohammad Hekmat-Nejad, Valery Polyakov, Richard Zang, Vijay Sethuraman, Payman Amiri, Mallika Singh, William R. Sellers, Emma Lees, Wenlin Shao, Michael P. Dillon, and Darrin D. Stuart. Design and discovery of n-(3-(2-(2-hydroxyethoxy)-6-morpholinopyridin-4-yl)-4-methylphenyl)-2-(trifluoromethyl)isonicotinamide, a selective, efficacious, and well-tolerated raf inhibitor targeting ras mutant cancers: The path to the clinic. *Journal of Medicinal Chemistry*, 63:2013–2027, 3 2020.

[51] Sandra Röhm, Benedict Tilman Berger, Martin Schröder, Apirat Chaikuad, Rob Winkel, Koen F.W. Hekking, Jorg J.C. Benningshof, Gerhard Müller, Roberta Tesch, Mark Kudolo, Michael Forster, Stefan Laufer, and Stefan Knapp. Fast iterative synthetic approach toward identification of novel highly selective p38 map kinase inhibitors. *Journal of Medicinal Chemistry*, 62:10757–10782, 12 2019.

[52] Hans Richter, Alexander L. Satz, Marc Bedoucha, Bernd Buettelmann, Ann C. Petersen, Anja Harmeier, Ricardo Hermosilla, Remo Hochstrasser, Dominique Burger, Bernard Gsell, Rodolfo Gasser, Sylwia Huber, Melanie N. Hug, Buelent Kocer, Bernd Kuhn, Martin Ritter, Markus G. Rudolph, Franziska Weibel, Judith Molina-David, Jin Ju Kim, Javier Varona Santos, Martine Stihle, Guy J. Georges, R. Daniel Bonfil, Rafael Fridman, Sabine Uhles, Solange Moll, Christian Faul, Alessia Fornoni, and Marco Prunotto. Dna-encoded library-derived ddr1 inhibitor prevents fibrosis and renal function loss in a genetic mouse model of alport syndrome. *ACS Chemical Biology*, 14:37–49, 1 2019.

[53] Kumiko Nagashima, Stuart D. Shumway, Sriram Sathyanarayanan, Albert H. Chen, Brian Dolinski, Youyuan Xu, Heike Keilhack, Thi Nguyen, Maciej Wiznerowicz, Lixia Li, Bart A. Lutterbach, An Chi, Cloud Paweletz, Timothy Allison, Youwei Yan, Sanjeev K. Munshi, Anke Klippel, Manfred Kraus, Ekaterina V. Bobkova, Sujal Deshmukh, Zangwei Xu, Uwe Mueller, Alexander A. Szewczak, Bo Sheng Pan, Victoria Richon, Roy Pollock, Peter Blume-Jensen, Alan Northrup, and Jannik N. Andersen. Genetic and pharmacological inhibition of pdk1 in cancer cells: Characterization of a selective allosteric kinase inhibitor. *Journal of Biological Chemistry*, 286:6433–6448, 2 2011.

[54] Thomas Hanke, Sebastian Mathea, Julia Woortman, Eidarus Salah, Benedict Tilman Berger, Anthony Tumber, Risa Kashima, Akiko Hata, Bernhard Kuster, Susanne Müller, and Stefan Knapp. Development and characterization of type i, type ii, and type iii lim-kinase chemical probes. *Journal of Medicinal Chemistry*, 65:13264–13287, 10 2022.
